# Autophagy Regulation by the Translation Machinery and Its Implications in Cancer

**DOI:** 10.3389/fonc.2020.00322

**Published:** 2020-03-13

**Authors:** Pilar Sarah Acevo-Rodríguez, Giovanna Maldonado, Susana Castro-Obregón, Greco Hernández

**Affiliations:** ^1^PSA-R and SC-O, División de Neurociencias, Instituto de Fisiología Celular, Universidad Nacional Autónoma de México (UNAM), Mexico City, Mexico; ^2^Translation and Cancer Laboratory, Unit of Biomedical Research on Cancer, National Institute of Cancer (Instituto Nacional de Cancerología, INCan), Mexico City, Mexico

**Keywords:** autophagy, translation initiation, cancer, mTOR, PERK, eIF2alpha, endoplasmic reticulum, ATG

## Abstract

Various metabolic pathways and molecular processes in the cell act intertwined, and dysregulating the interplay between some of them may lead to cancer. It is only recently that defects in the translation process, i.e., the synthesis of proteins by the ribosome using a messenger (m)RNA as a template and translation factors, have begun to gain strong attention as a cause of autophagy dysregulation with effects in different maladies, including cancer. Autophagy is an evolutionarily conserved catabolic process that degrades cytoplasmic elements in lysosomes. It maintains cellular homeostasis and preserves cell viability under various stress conditions, which is crucial for all eukaryotic cells. In this review, we discuss recent advances shedding light on the crosstalk between the translation and the autophagy machineries and its impact on tumorigenesis. We also summarize how this interaction is being the target for novel therapies to treat cancer.

## Introduction

Cancer often results from glitching the interconnection between different metabolic networks and molecular processes (1), such as translation and autophagy. Translation is a fundamental process for all forms of life because it plays a central role in gene expression, and translational control critically contributes to the composition and quantity of a cell's proteome (2–5). Recently, dysregulation of translational control has been recognized as a cause of malfunctioning of other key cellular processes, which may lead to the onset and development of different types of cancer (6–10). Here, we discuss current research shedding light on the interplay between translation and autophagy and its involvement in cancer. We finally discuss new drugs targeting these processes to treat this malady.

## Translation Initiation and Its Regulation

### An Overview

Translation consists of initiation, elongation, termination, and a final stage of ribosome recycling that drives to a new round of translation. It is one of the most energy-consuming process in the cell. The whole process is largely controlled at the initiation step and, in consequence, defects in the translation initiation machinery or the signaling pathways regulating this step have different consequences on the cell that lead to numerous diseases, including cancer (11, 12).

**Figure 1:**
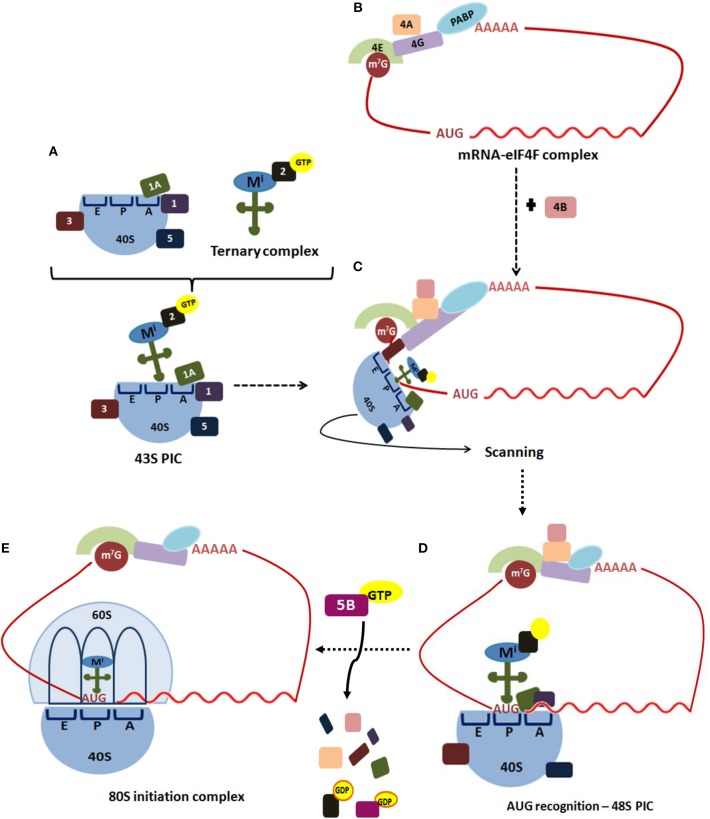
Translation initiation in eukaryotes. Translation of most eukaryotic mRNAs is mediated by the eukaryotic initiation factors (eIFs). **(A)** This process begins when the free 40S ribosomal subunit, which is stabilized by eIF3 *(3)*, eIF1 *(1)*, eIF1A *(1A)*, and eIF5 *(5)*, binds to a ternary complex consisting of eIF2-GTP bound to an initiator Met-tRNAi, forming 43S pre-initiation complex *(PIC)*. **(B)** Simultaneously, the cap structure *(m*^7^*G)* located at the 5′-end of an mRNA is recognized by the cap-binding protein, eIF4E *(4E)*. The scaffold protein eIF4G *(4G)* performs simultaneous interactions with the cap-bound eIF4E, the ATP-dependent RNA-helicase eIF4A *(4A)* and PABP bound to poliA, circularizing the mRNA to form the mRNA-eIF4F complex. **(C)** The ribosome-bound eIF3 coordinates the recruitment of the 43S PIC to the mRNA 5'-UTR. The 43S PIC scans base-by-base the mRNA 5′-UTR to reach the AUG start codon, a process in which eIF4A, assisted by eIF4B *(4B)*, unwinds secondary structures of the 5′-UTR. **(D)** Selection of the start codon establishes the open reading frame for mRNA decoding, and results in a 48S PIC with the ${\text{Met}-\text{tRNA}}_{\text{i}}^{\text{Met}}$ and eIF1A tightly positioned within the P-site. **(E)** Then, a GTP-eIF5B complex promotes release of eIF1 and eIF5B, facilitating joining of a 60S ribosomal subunit to the 48S PIC to assemble an 80S initiation complex, which is ready to start the elongation step of translation.

The initiation step of translation consists in the recruitment of the small (40S) ribosome subunit to the 5′-UTR (see Table 1 for abbreviations) of an mRNA and the selection of the translation start site, usually an AUG codon (depicted in Figure 1) (5, 13, 14). Translation initiation starts when the cap structure (m^7^GpppN, where N is any nucleotide) located at the 5′-end of an mRNA is recognized by the cap-binding protein, the eukaryotic initiation factor (eIF) 4E (Figure 1). In a parallel set of reactions, a free 40S ribosomal subunit interacts with eIF1, eIF1A, eIF3, eIF5, and a ternary complex (consisting of eIF2 bound to GTP and an initiator ${\text{Met}-\text{tRNA}}_{\text{i}}^{\text{Met}}$) to form a 43S pre-initiation complex (PIC). This step loosely positions the initiator ${\text{Met}-\text{tRNA}}_{\text{i}}^{\text{Met}}$ in the peptidyl (P) decoding site of the ribosome.

**Table 1:** Abbreviations.

**Abbreviation**	**Definition**
3′-UTR	3′ untranslated region
5′-UTR	5′ untranslated region
4E-BPs	eIF4E-binding proteins
Akt	Protein kinase B
AMPK	Adenosine monophosphate–activated protein kinase
ATF4	Activating Transcription Factor 4
Atg	Autophagy related genes
BECN1	Beclin-1
CHOP	C/EBP Homologous Protein
DDX6	DEAD-Box Helicase 6
DEPTOR	DEP domain-containing mTOR-interacting protein
Dhh1	DExD/H-box helicase
eEF2K	elongation factor 2 kinase
eIF	eukaryotic initiation factor
GABARAP	Gamma-aminobutyric acid receptor-associated protein
GCN2	General control non-repressed 2 kinase
hnRPA1	Heterogeneous Nuclear Ribonucleoprotein A1
HRI	Heme-regulated inhibitor
Hu	Human antigen
LC3	Microtubule-associated protein 1A/1B-light chain 3
MAPK	Mitogen-activated protein kinases
mLST8	mammalian lethal with SEC13 protein 8
mSIN1	mammalian stress-activated protein kinase interacting protein 1
mTOR	mammalian/mechanistic target of rapamycin
mTORC1	mammalian target of rapamycin complex 1
mTORC2	mammalian target of rapamycin complex 2
Orb	Oo18 RNA-binding protein
p62/SQSTM1	p62/Sequetosome1
PABP	Poly(A)-binding protein
PDCD4	Programmed cell death 4 protein
PERK	PKR-like endoplasmic reticulum kinase
PI3K	Phosphatidylinositol 3-kinase
PI3KC3/VPS34	Class III Phosphatidylinositol 3-kinase
PIC	Pre-initiation complex
PIK3R4	Phosphoinositide 3-kinase regulatory subunit 4
PKR	Double-stranded RNA activated protein kinase
PRAS40	Proline-rich Akt substrate 40 kDa
PROTOR	Protein observed with RICTOR
Psp2	Polymerase suppressor protein 2
PtdIns3P	Phosphatidylinositol 3-phosphate
RACK1	Receptor for activated C kinase 1
RAPTOR	Regulatory associated protein of mTOR
RHEB	Ras homolog enriched in brain
RICTOR	Rapamycin-insensitive companion of TOR
RPS27L	Ribosomal protein S27-like
S6Ks	Protein kinases S6 kinases
TSC1/2	Tuberous sclerosis complex 1/2
ULK1/2	Unc51-like kinase 1/2
WIPI	WD-repeat protein interacting with phosphoinositides
ZFP36/TTP	Zinc finger protein 36 homolog/Tristetraprolin

The scaffold protein eIF4G performs simultaneous interactions with the cap-bound eIF4E, the ATP-dependent RNA-helicase eIF4A, the poly(A)-binding protein (PABP) and the ribosome-bound eIF3, to coordinate recruitment of the 43S PIC to the mRNA 5′-UTR. Afterward, 43S PIC scans base-by-base the mRNA 5′-UTR to reach the AUG start codon, a process in which eIF4A, assisted by eIF4B, unwinds secondary structures of the 5′-UTR. Fidelity in the recognition of the correct mRNA AUG start codon is driven by eIF1 and eIF1A, which stabilize Watson-Crick base-pairing between the AUG codon and the ${\text{Met}-\text{tRNA}}_{\text{i}}^{\text{Met}}$ CAU anticodon. Selection of the start codon establishes the open reading frame for mRNA decoding, and results in a 48S PIC with the ${\text{Met}-\text{tRNA}}_{\text{i}}^{\text{Met}}$ and eIF1A tightly positioned within the P-site. Then, a GTP-eIF5B complex promotes release of eIF1 and eIF5B, facilitating joining of a 60S ribosomal subunit to the 48S PIC to assemble an 80S initiation complex, which is ready to start the elongation step of translation (13–15).

Ribosomal proteins, RNA binding proteins and miRNAs regulate protein synthesis either targeting global mRNAs by inhibiting or activating general translational machinery, or targeting specific mRNAs. Although this type of regulation can take place at initiation, elongation, and termination of translation, the rate-limiting step is initiation, and hence the most common and effective target (13–15).

### Regulation of Translation Initiation

Different signaling cascades control protein synthesis in response to various stimuli, such as the MAPK pathway and the PI3K/Akt/TSC/RHEB/mTORC1 pathway (16, 17). A third pathway also regulates translation at the initiation step via phosphorylation of the eIF2 alpha subunit at Ser51 by four different protein kinases detailed below (18, 19). The MAPK pathway was not considered in this review.

**Figure 2:**
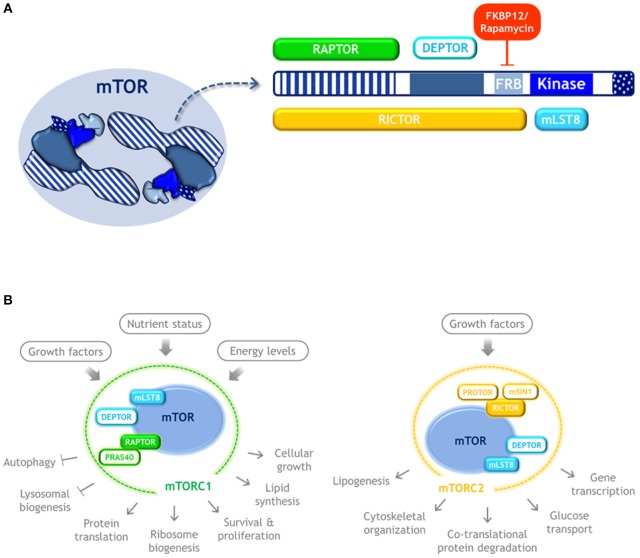
mTOR kinase structure and complexes. **(A)** Schematic representation of mTOR kinase domains and its interacting proteins. mTOR possess 5 main domains (highlighted in blue). As an active form, mTOR dimerizes and may form two distinct complexes. mTORC1 is composed by three subunits that cooperate to phosphorylate substrates: mTOR, RAPTOR, and mLST8, and by the inhibitory subunits DEPTOR and PRAS40. Rapamycin forms a complex with FKBP12 that binds to mTOR and inhibits mTORC1 signaling. mTORC2 also contains mTOR, DEPTOR, and mLST8, but instead of RAPTOR it contains RICTOR, as well as the regulatory subunits mSIN1, and PROTOR. **(B)** mTORC1 and mTORC2 respond to distinct stimulus and control different cellular process. Color code: blue, mTOR kinase; cyan, components of both mTOR complexes; green, MTORC1 exclusive components; yellow, MTORC2 exclusive components.

mTOR is a serine/threonine kinase that dimerizes and forms the catalytic subunit of two functionally distinct multiprotein complexes, namely mTORC1 and mTORC2 (16, 17, 20–22) (Figure 2). mTORC1 is composed by three subunits that cooperate to phosphorylate substrates: mTOR itself, RAPTOR and mLST8; and by two inhibitory subunits: DEPTOR and PRAS40. The mTORC1 signaling pathway senses nutrient availability, growth factors, and cellular energy levels to promote cellular growth, survival, and proliferation, as well as translation, ribosome biogenesis, and lipid synthesis. It also blocks key catabolic processes such as autophagy and lysosome biogenesis. It is sensitive to rapamycin, a compound that forms a gain of function complex with the peptidyl-prolyl isomerase FKBP12 that binds to mTOR and inhibits mTORC1 signaling. Therefore, rapamycin is an inducer of autophagy.

**Figure 3:**
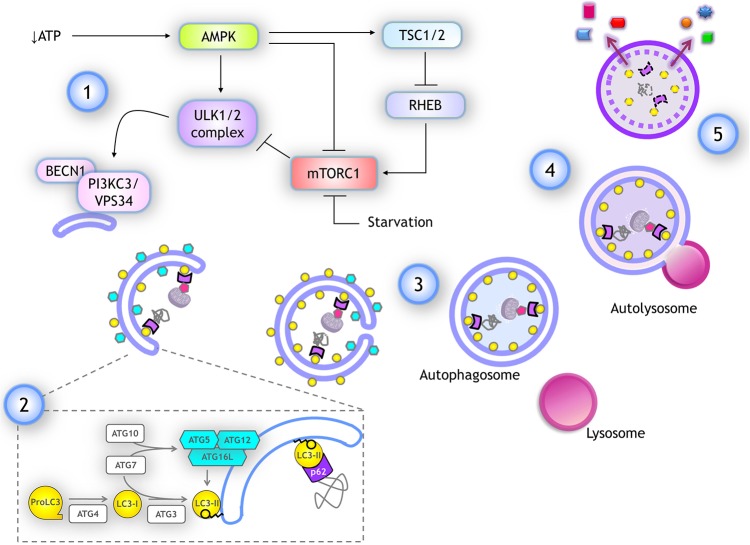
Autophagy: overview and key molecular components. (1) Several stimuli promoting autophagy, like a drop in ATP, lead to AMPK activation, which stimulates autophagy by activating ULK1/2 complex and inhibiting mTORC1 through TSC1/2 activation, which in turn inactivates RHEB, a negative regulator of mTORC1. ULK1/2 complex activates the class III PI3K complex I by phosphorylating PIK3C3/VPS34. (2) For phagophore elongation, conjugation of ATG5-ATG12 complex is catalyzed by ATG7 and ATG10. ATG5-ATG12 covalently linked then interact with ATG16L forming a complex that is recruited at the phagophore. LC3 is proteolytically cleaved upon its translation by ATG4, producing LC3-I form. When autophagy is induced, LC3-I is covalently bound to phosphatidylethanolamine (PE) at the membrane of the phagophore. This reaction is catalyzed by ATG3 (E1-like) and ATG7 (E2-like) again, while ATG12-ATG5/ATG16L complex already recruited at the phagophore surface functions as an E3-like enzyme. Lipidated LC3-I is named LC3-II and it remains anchored to the elongating phagophore. LC3-II associates to both inner and outer membranes of the phagophore in expansion. Cargo is recognized by adaptor proteins like p62/SQSTM1, which also binds to LC3-II. (3) After elongation is completed the tips of the vesicle fuse giving rise to a double membrane vesicle named autophagosome. Autophagosomes maintain LC3-II at the inner membrane. (4) Autophagosomes fuse with lysosomes and the autophagosome inner membrane is degraded with the cargo, LC3-II, and adaptor proteins. (5) Finally, some of the products of degradation could be recycled, being released back into the cytoplasm.

When amino acids are abundant, mTORC1 stimulates protein translation and inhibits autophagy by phosphorylating ULK1/2 at S757 and S637 residues, resulting in its catalytic activity suppression. Phosphorylation in these sites also disrupts the interaction of ULK1 with AMPK (23), a kinase activated by low glucose and ATP levels, and is the main activator of autophagy. AMPK activates ULK1 by phosphorylation at different serine residues (24), and inactivates mTORC1 phosphorylating RAPTOR and indirectly, by activating TSC2 (which in turn inhibits RHEB, a mTOR activator) (25) (schematized in Figure 3). When the amino acids pool is reduced, mTOR is inactivated allowing ULK1 dephosphorylation by the PP2A-B55α complex (26), while upon autophagy induction by genotoxic agents, ULK1 is dephosphorylated by PPM1D phosphatase (27).

mTORC2 also contains mLST8 and DEPTOR, but instead of RAPTOR it contains RICTOR, as well as mSIN1, and PROTOR. mTORC2 regulates co-translational protein degradation, lipogenesis, glucose transport, gene transcription, and cytoskeletal organization (21, 22). Since mTORC1 is the one involved in the control of translation and autophagy, only mTORC1 is further reviewed here.

To regulate protein synthesis, mTORC1 phosphorylates eIF4E-binding proteins (4E-BPs) that directly regulate eIF4E: hypophosphorylated 4E-BPs bind eIF4E with high affinity, which precludes eIF4E association with eIF4G, thus repressing cap-dependent translation. On the contrary, the hyperphosphorylated species of 4E-BPs dissociate from eIF4E to relieve translational repression. mTORC1 also phosphorylates S6Ks and eEF2K, that phosphorylate translation factors eIF4B, eIF4G, elongation factor eEF2, the ribosomal protein S6 and PDCD4, a negative regulator of eIF4A (16, 17, 20).

eIF2 phosphorylation at the alpha subunit is a key mechanism to regulate translation initiation. Upon mRNA AUG start codon recognition by the ribosome, ternary complex GTP/eIF2/${\text{Met}-\text{tRNA}}_{\text{i}}^{\text{Met}}$ delivers ${\text{methionyl}-\text{tRNA}}_{\text{i}}^{\text{Met}}$ to the ribosomal P-site to arrest scanning, form the 80S Initiation Complex, and further initiates mRNA decoding. eIF2alpha activity relays on its phosphorylation status: whereas non-phosphorylated eIF2alpha promotes translation, phosphorylated eIF2alpha at Ser51 binds with high affinity to the guanine nucleotide exchange factor eIF2B, leading to the formation of inactive eIF2B–eIF2–GDP complex that represses global translation. Upon diverse stimuli, mammalian eIF2alpha can be phosphorylated by four stress-responsive protein-serine/threonine kinases, namely PKR, that responds to virus infection; GCN2, that becomes activated in response to amino acids depletion, UV radiation, high salinity, and viral infection; HRI, that responds to oxidative agents, heat shock, and heme groups deficiency; and PERK, a transmembrane protein that becomes activated in response to perturbations in endoplasmic reticulum and unfolded proteins (18, 19).

## Autophagy

### An Overview

Autophagy is mainly a catabolic process that delivers cytoplasmic components for lysosomal degradation. In mammals, there are three pathways to deliver the cargo into the lysosomes: (1) Macroautophagy, where cargoes are first recognized and engulfed into a specialized double-membrane vesicle termed the “autophagosome.” Afterward, it fuses with lysosomes to create the “autolysosome.” This review focuses on this mechanism, which for simplicity will be referred to as “autophagy.” Other mechanisms delivering cytoplasmic material into lysosomes are (2) Chaperone-mediated autophagy, where specific proteins are translocated into the lysosome; and (3) Endosomal microautophagy, where cytoplasmic cargoes get engulfed directly by late endosomes or multivesicular bodies. The latter processes have been revised elsewhere (28).

Autophagy can degrade all kind of macromolecules, whole organelles, and even intracellular pathogens. The physiological function of autophagy depends on the inducer and the fate of the degraded cargo. Autophagy is not merely a catabolic process but rather functions as a metabolic integrator, sometimes inducing anabolism. For instance, under a lack of nutrients, autophagy is triggered to degrade long-lived proteins for amino acids recycling for the synthesis of essential proteins; lipid droplets can also be degraded to release free fatty acids or even glycogen is degraded to release glucose, hence fostering anabolic biochemical pathways (29). Cancerous cells in solid tumors benefit from these functions, as autophagy allows them to resist under low oxygen and nutrients availability, maintaining the metabolic pathways necessary for aggressive tumor growth (30). Autophagy is also induced in response to several stressors, such as genotoxic compounds. In this case, autophagy maintains genome integrity and consequently, autophagy malfunctioning leads to tumorigenesis (31). Nevertheless, autophagy plays a dual role in cancer, as some cancerous cells acquire chemotherapy resistance through activating autophagy (32). Since autophagy prevents early tumor formation but also is able to promote tumor cells survival, more comprehensive understanding of the autophagy involvement in carcinogenesis is needed before a therapy can be established.

### Molecular Mechanisms of Autophagy

The regulation and execution of autophagy are mediated by several proteins known as ATG (autophagy related) (33). Here, we review only key proteins whose mRNAs are a target for translational regulation. The process of autophagy is divided into five steps (an overview is depicted in Figure 3):

1. *Initiation*. Upon autophagy induction, the ULK1/2 complex is activated. It is composed of ATG13, RB1CC1 and ATG101. ULK1/2 is a serine/threonine kinase that phosphorylates and activates the Class III PI3K complex I (composed of PIK3C3/VPS34, BECN1, PIK3R4, ATG14). This complex generates PtdIns3P at the surface of the membrane where the phagophore will form, most commonly at the endoplasmic reticulum membrane. PtdIns3P recruits WIPI family proteins, setting up the site of nucleation to further recruit molecules that give rise to the autophagosome.
2. *Elongation*. Two ubiquitin-like complexes are conjugated to promote phagophore elongation around the engulfed cargo. The first conjugation forms the ATG12-ATG5 complex. ATG12 is a small protein with structural similarity to ubiquitin, which is covalently bound to ATG5 by ubiquitin-like biochemical reactions catalyzed by ATG7 (E1-like) and ATG10 (E2-like) enzymes. This complex seems to be constitutively formed after ATG5 and ATG12 translation. When autophagy is induced, ATG12-ATG5 complex interacts with several molecules of ATG16L, forming a multiprotein complex that is recruited to the phagophore. Separately, upon its translation, protein LC3 (encoded by *MAP1LC3B* gene) is cleaved by the protease ATG4, producing the LC3-I isoform. When autophagy is induced, LC3-I is covalently bound to phosphatidylethanolamine at the phagophore's membrane. This reaction is catalyzed again by ATG7 (E1-like) and by ATG3 (E2-like), while ATG12-ATG5/ATG16L complex already recruited to the phagophore surface functions as an E3-like enzyme. Lipidated LC3-I is termed LC3-II and it remains anchored to the elongating phagophore.
  Detecting LC3-II abundance is a common way to monitor autophagy induction. It is also common to follow intracellular localization of GFP-LC3, since the unlipidated form (corresponding to LC3-I) is diffused in the cytoplasm. As it gets lipidated and anchored to the phagophore (corresponding to LC3-II), upon autophagy induction, LC3-II displays a punctuated pattern when observed by fluorescence microscopy.
  Cargo recognition occurs during phagophore elongation. Cytoplasmic material to be degraded is labeled by specific proteins, such as ubiquitin. Adaptor proteins serve as autophagic receptors to bridge the labeled cargo with the surrounding phagophore, leading the direction of membrane elongation around the cargo. Autophagic receptors have a domain to interact with the protein label and another domain to interact with LC3-II (or members of the family LC3/GABARAP) at the phagophore's membrane. The most common autophagic receptor is p62/SQSTM1.
3. *Closure*. Phagophore continues elongating around the cargo until its tips fuse, giving rise to the double-membrane vesicle termed autophagosome. Other proteins and lipids contribute to the autophagosome closure and have been recently reviewed (34). Once autophagosome forms, LC3-II is detached from the outer membrane giving rise to a mature autophagosome, ready to fuse with a lysosome (35).
4. *Fusion*. Autophagosomes travel through microtubules to reach and fuse with lysosomes, giving rise to autolysosomes. The molecular machinery for autolysosomes fusion has been recently reviewed (34).
5. *Degradation and recycling*. Within the autolysosomes, lysosome hydrolases digest cytoplasmic cargoes, the inner membrane of the autophagosome, and associated proteins like LC3-II and p62/SQSTM1 as well. If autophagy was induced by a lack of nutrients, macromolecules building blocks are released into the cytoplasm through specific transporters and permeases that are recruited during autophagosome formation. Then, the lysosome membrane segregates in the autolysosome and elongates until a new lysosome is detached and reconstituted (34). The fate of the remaining autolysosome is poorly understood.

When analyzing autophagy, it is essential to study not only the accumulation of LC3-II or autophagosomes, but also to verify the cargo degradation. An increase in the abundance of LC3-II, for example, could be a consequence of an interruption of the autophagic flux instead of a true autophagic induction. The most common way to evaluate the autophagic flux is by comparing the abundance of an autophagic adapter such as p62/SQSTM1 or verifying cargo degradation. For a full description of methods to monitor autophagy see (36).

In the next section, we review how translation machinery modulates autophagy in normal and cancerous cells.

## Regulation of Autophagy by Translation

**Figure 4:**
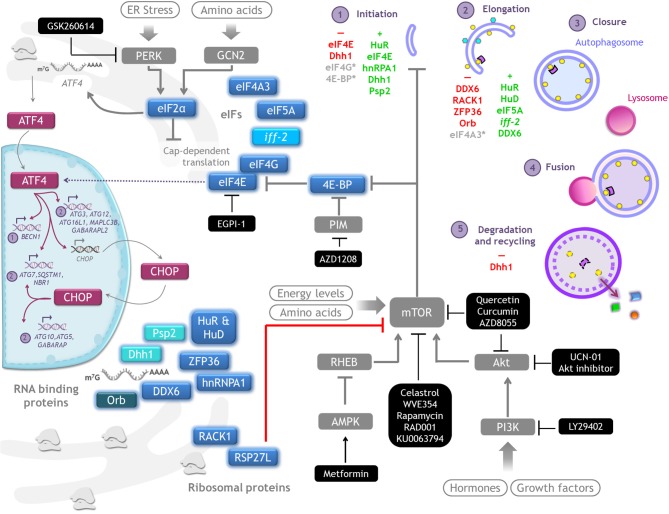
Autophagy regulation by translation machinery, and therapeutics targets. Integrative scheme of the examples of autophagy regulation described on conditions found in tumor environment such as hypoxia, starvation, or cell death resistance. Although the main control of autophagy occurs at translational level, eIF4E and eIF2alpha are able to regulate the transcription of some *ATG* genes through ATF4/CHOP. Color code: *magenta*, transcriptional regulators of *ATG* genes; blue, proteins that control translation of *ATG* mRNAs (a different intensity of blue denotes observations made on different species); gray, signaling pathways upstream of autophagy. Therapeutics agents against cancer targeting key molecules for protein translation and autophagy regulation are shown in black boxes.

Since its discovery, autophagy regulation has been broadly studied with focus on understanding *ATG* genes transcriptional regulation and ATG proteins post-translational modifications. In recent years, however, a new level of integration of information has emerged: the post transcriptional regulation of *ATG* mRNAs expression by the translation machinery. Here we summarize investigations that use gain- or loss- of-function approaches to learn about the regulation of *ATG* mRNAs translation by eIFs, ribosomal proteins and RNA binding proteins, and how these interventions affect autophagy (Figure 4). It is important to consider that in several of these studies only LC3-II or autophagosomes abundance were studied, without distinguishing whether there was an autophagic flux blockage or a true autophagy induction. In those cases, it is not possible to conclude that a functional autophagy takes place. We review in Table 2 specific experiments performed to analyze autophagy.

**Table 2:** *ATG* mRNAs expression regulated by translation machinery.

**Protein Studied**	**Model**	**Autophagy evaluation**	**Autophagy flux assessment**	**Additional observations**	**References**
4E-BP1	LHMB-AR PrEC sh4E-BP1	↑LC3-II, ↑acridine orange			(37) #122
4E-BP1	HL60 or HeLa parthenolide HEK293 Parthenolide + sh4E-BP1 HeLa Parthenolide + plasmid 4E-BP1	↑LC3-II, ↑GFP-LC3 ↑LC3-II ↓LC3-II		↓ 4EB-P1 ↓Ⓟ 4E-BP1	(38) #127
4EB-P1	HCT-1116 hypoxia	↑ translation 35 lysosomal mRNAs ↑ acridine orange ↑ LysoTracker^+^ ↑ LC3-II ↓p62		↑Ⓟ eIF2α; ↓Ⓟ 4EB-P1 ↑ translation *EIF4EBP3* and *EIF2AK3*; *EIF4E, RPS6K* subunits	(39) #40
4E-BP1 Akt S6K1	HepG2.2.15 si4E-BP1 siAkt or Akt inhibitors siS6K1	↑LC3-II ↓LC3-II ↓GFP-LC3 puncta ↑p62 ↓LC3-II	CQ ↑ LC3-II CQ ≠ LC3-II		(40) #161
eIF4E	T-ALL Jurkat selenite selenite +sieIF4E	↑LC3-II ↑GFP-LC3 puncta ↑ATF4 on *MAP1LC3B* and *CHOP* promoters ↓GFP-LC3 puncta ↓ATF4 on *MAP1LC3B* and *CHOP* promoters	Baf A1 ↑LC3-II ↑p62	↑CHOP, ↑Ⓟ eIF4E ↓ATF4 + si_p38 or p38 inhibitors prevent selenite effects	(41) #124
eIF4E eIF2α	NB-4 selenite selenite+ sieIF2α selenite+ plasmid eIF4E	↓LC3-II, ↑p62 ↓GFP-LC3 puncta ↓ATF4 on *MAP1LC3B* promoter ↑LC3-II, ≠GFP-LC3 ↑LC3-II ↑GFP-LC3 puncta ↑ATF4 on *MAP1LC3B* promoter		↑CHOP, ↑ATF4 ↑Ⓟ eIF2α, ↓Ⓟ eIF4E ↓CHOP ↑CHOP	(42) #43
eIF5A eIF4A3	MCF-7 sieIF5A sieIF5A +Torin-1	↓GFP-LC3 puncta ↓autophagosome (TEM) ↓ATG3 ↓GFP-LC3 puncta	Baf A1 ↑LC3-II		(43)
*iff-2* (eIF5A homlog)	*C. elegans iff-2* RNAi	↓GFP::LGG-1 puncta			(43)
eIF4G1 eIF4G2	MCF10A or HEK293T sheIF4G1 or sh eIF4G2	↑LC3-II ↑GFP-LC3 puncta ↑MDC^+^ Vesicles			(44) #216
eIF4G1	MCF10A sheIF4G1 γ irradiation	↑LC3-II ↑GFP-LC3 puncta			(45) #2
RACK1	HT1080 siRACK1 HepG2, Hep3B, U2OS, HeLa, MCF-7 and MDAMB231	↑LC3-II ↑LAMP1 and LAMP2 ↑GFP-LC3 puncta and colocalization with LysoTracker ↑BCL-XL and BECN1 interaction ↓p62 ↑polysomal fraction on *MAP1LC3* and *BCL-XL* mRNA	Baf A1 ↑LC3-II ↑p62		(46) #126
RPS27L	MB231 or SK-BR3+ siRPS27L	↑LC3-II ↑EGFP-LC3 puncta ↓p62	+CQ or Baf A1: ↑LC3-II and ↑p62	↑DEPTOR, ↓Ⓟ S6K1 and Ⓟ 4EBP1 ↓Ⓟ S6K1 and Ⓟ 4EBP1	(47) #131
	MEFs *RSP27L^−/−^*	↑LC3-II ↓p62			
HuD	βTC6 or U2OS siHuD plasmid HuD	*≠ATG5 mRNA*; ↓ATG5 ↓LC3-II ↓GFP-LC3 puncta ↓autophagosomes (TEM) *≠ATG5 mRNA* ↑ATG5 ↑LC3-II ↑GFP-LC3 puncta		+miR-181 ↓EGFP-*3'UTR Atg5 mRNA* ↑EGFP-*3'UTR Atg5 mRNA*	(48) #125
HuR	HSC-LX2 or HSC-T6 erastin+ siHuR HSC-LX2 or HSC-T6 erastin+ plasmid HuR	↓LC3-II ↓BECN1 ↑p62 ↑LC3-II ↑BECN1 ↑autophagosome (TEM), ↓p62	CQ: ↑LC3-II	RIP: *3'UTR BECN1 mRNA* enrichment	(49)
HuR	L-02 or Hep3B siHuR	↓ATG5, ATG12 and ATG16 ↓polysome association to *ATG5, ATG12 and ATG16 mRNAs* ↓LC3-II ↓Autophagosome and autolysosome (TEM) ↓GFP-LC3 puncta	colchicine modest ↑LC3-II	RIP: *3'UTR ATG5, ATG12, ATG16 mRNAs* enrichment	(50) #36
HuR	HK-2 hypoxia HK-2 hypoxia+ shHuR	↑LC3-II, ATG7 and ATG16 ↓LC3-II, ATG7 and ATG16		↑TUNEL^+^ cells RIP: *ATG7, ATG16 mRNAs* enrichment	(51)
HuR	MCF-7 starvation MCF-7, MDA-MB 231, PC3, HaCat siHur	↑LC3-II, ↑BECN1, ↑polysomes association of *BECN1 mRNA* & HuR, ↓LC3-II ↓BECN1 ↓*BECN1 mRNA*		RIP: *3'UTR BEC1 mRNA* enrichment	(52)
HuR	Intestinal epithelium IE_HuR^−/−^ mice	↓LC3-II ↓BECN1, ↓ATG16L1 ↓ATG7		RIP: *ATG16 mRNA* enrichment	(53)
	IECs siHuR	↓LC3-II ↓ATG16L1 ↓ newly synthesized ATG16L1			
ZFP36	HSC-LX2 or HSC-T6 erastin + plasmid ZFP36	↓ LC3-II ↓GFP-LC3 puncta ↓ ATG16L1 ↓ ATG5-ATG12 ↓ Autophagosome (TEM) ↓*ATG16 mRNA*, ↑*SQSTM11 mRNA*		RIP: *ATG16 mRNA* enrichment Luc-*3'ÚTR Atg16 mRNA*: ↓Luc activity	(54)
	HSC-LX2 or HSC-T6 erastin + plasmid FXBW7	↑ LC3-II, ↑ ATG16L1, ↑ ATG5-ATG12 ↑ Autophagosome (TEM)			
hnRNPA1	HCT-116 sihnRNPA1 plasmid hnRNPA1	↓BECN1, ≠ *Becn1 mRNA* ↑BECN1, ≠ *Becn1 mRNA*		Luc-*3′ÚTR Becn1 mRNA*: ↓Luc activity Luc-*3′ÚTR Becn1 mRNA*: ↑ Luc activity Biotin-*3′UTR Benc1 mRNA* RIP: *Becn1 mRNA* enrichment	(55)
Orb	*Drosophila* germarium Orb mutant	↑*Atg12 (mRNA);* ↑Atg12 and Atg8 (protein); ↑LysoTracker^+^ structures		RIP: *Atg12 mRNA* enrichment	(56) #8
Dhh1 (DDX6)	Yeast Δd*hh1* nutrient replete	*↑ Atg3, Atg7, Atg8, Atg19, Atg20, Atg22 and Atg24* mRNA	GFP- ATG8 processing assay Δd*hh1+ starvation*: ↑ GFP free		(57) #123
	Mouse ESC DDX6 ^+/−^	*↑Map1lc3* mRNA ↑LC3-II ↓LC3 puncta ↓p62			
	HeLa+ siDDX6	*↑ MAP1LC3* mRNA ↑ LC3 puncta			
	HeLa+ plasmid DDX6	↓*MAP1LC3* mRNA ↓ LC3-II ↓LC3 puncta ↑p62			
Dhh1 (DDX6) Eap1	Yeast Δ*dhh1* Nitrogen starvation HEK293A DDX6 ^−/−^ Amino acid starvation	↓Atg1, Atg13 (protein) ≠ *ATG1 and ATG13* mRNAs ↓ATG16L1 ↑*ATG16L1* mRNA	Pgi-GFP processing assay: ↓ free GFP		(58) #24 ó 1
Psp2	Yeast Δ*psp2*+ Nitrogen starvation	↓Atg1 ≠ *ATG1* mRNA ↓polysomes association of *ATG1* mRNA, ↓Atg13	Pgi-GFP processing assay: ↓ free GFP Pho8Δ60 assay: ↓ vacuolar Pho8Δ60	RIP: *ATG1 & ATG13* mRNA enrichment	(59) #274
ATF4 CHOP	MEFs *ATF4 ^−^*^/−^ leucine starvation	≠ *Atg16l1, Map1lc3b, Atg12, Atg3, Becn1, Gabarapl2, p62, Nbr1, Atg7* mRNAs		↑Ⓟ eIF2α	(60) #3
	MEFs *CHOP ^−^*^/−^ leucine starvation	≠ *Atg10, Gabarap, Atg5, p62, Nbr1, Atg7 mRNAs*		↑Ⓟ eIF2α	
PERK ATF4	LNCaP Tunicamycin	↑ LC3-II mTagRFP-mWasabi-LC3: ↑ red punctate ↑*MAP1LC3B, GABARAPL1, WIPI1, MAPLC3B2, MAPLC3A, ATG13, ATG16L1, GAGARAP, ATG12, ATG5, ATG3, BECN1* mRNA	BafA1: ↑ LC3-II mTagRFP-mWasabi-LC3: ↑ yellow puncta LDH sequestration assay: ↑ sequestration rate LLPD assay: ↑ Valine release		(61) #128
	LNCaP Tunicamycin siATF4	↓*MAP1LC3B, GABARAPL1, WIPI1, MAPLC3B2, MAPLC3A, ATG13* mRNA	↓↓ Valine release		
eIF2α ATF4	MEFs eIF2Aα non-phosphorylable mutant or *ATF4^−^*/^−^ Rapamycin	↓*Map1lc3 and Atg5* mRNA ↓LC3-II and LC3 puncta	GFP-LC3 processing assay: ↓ free GFP		(62) #130

### Translation Initiation Factors Control Autophagy

In vertebrates, the family of 4E-BPs contains three members: 4E-BP1, 4EB-P2, and 4E-BP3, and all of them function as repressors of cap dependent translation by sequestering eIF4E thus preventing its interaction with eIF4G (14). Among them, 4E-BP1 is the best characterized. The first study that suggested an inhibition of autophagy by 4E-BP1 was done in genetically engineered immortalized and tumorigenic human prostate epithelial cells (PrEC) that overexpressed *MYC* oncogene. MYC binds to the regulatory region of *4EBP1* gene increasing its expression, which leads to a decreased autophagy. The inhibitory role of 4E-BP1 over autophagy was confirmed by the observation that cells with reduced expression of 4E-BP1 accumulate autophagosomes (37). A negative regulation of this translation repressor over autophagy is also true in human hepatoblastoma cells with stable expression of hepatitis B virus (HepG2.2.15), since again, silencing 4E-BP1 expression increases LC3-II, and blocking autophagic flux with chloroquine results in an even greater accumulation, indicating that LC3-II accumulates due to an activation of autophagy (40).

Tumor cells have to adapt to hypoxia by altering their gene expression and protein synthesis; while general translation is inhibited, selected mRNAs remain efficiently translated. A study searching for such hypoxia-regulated genes found translational up-regulation of lysosomal proteins in human colon cancer cells, associated with 4EB-P1 dephosphorylation. The study of autophagy induction was more complete in this work, since in addition of detecting an increased number of autophagosomes, they also found more autolysosomes and lysosomes, as well as a decrease of the adaptor protein p62/SQSTM1, demonstrating the autophagic flux is not interrupted (39). However, further experiments are necessary to elucidate the mechanisms by which 4E-BP1 inhibits autophagy, since Lan et al. found that neither its phosphorylation nor its binding to eIF4E are necessary for the regulation of autophagy (38). Nevertheless, the relevance of 4E-BPs phosphorylation should not yet be ruled out since in the cited work only two out of seven phosphorylation sites were mutated, and other kinases additional to mTOR could also phosphorylate 4E-BPs (63). An alternative mechanism for the negative effect of 4E-BP1 over autophagy could be by stabilization of the mTORC1-ATG13-RB1CC1 complex, leading to autophagy repression at the initiation step. Interestingly, it has recently been described in yeast a repression of translation role for the eIF4E-interacting protein p20 in an eIF4E-independent manner, where p20 remains bound to its mRNAs targets (64).

eIF4E is a key component of the eIF4F complex, and its level and availability limit the translation process. eIF4E phosphorylation is important to promote selective translation of a subset of mRNAs related to proliferation, inflammation, and survival (7). Since autophagy contributes to mitigate various types of stress to avoid cell death, *ATG* mRNAs might belong to the subset of selected mRNAs translated when global translation is inhibited.

#### Transcriptional Control of Autophagy Mediated by Translation Initiation Factors

During Unfolding Protein Response PERK phosphorylates eIF2alpha, leading to global protein translation shut down but allowing *ATF4* translation. ATF4 is a transcription factor that upregulates expression of stress-responsive genes, including *ATG* genes and *CHOP*. CHOP, likewise, plays a critical role in adaptation to stress and also induces transcription of some *ATG* genes, while a subset of genes needs to be upregulated by both ATF4 and CHOP (60), possibly to ensure a rapid stress relief.

eIF4E regulates ATF4 binding to some promoters, being *MAP1LC3B* (gene coding for LC3) among them. A couple of observations studying different leukemia cell lines suggest that eIF4E can be both a negative and a positive regulator of autophagy by modulating the transcription of *MAP1LC3B*, apparently depending on the function of the tumor suppressor p53 (41, 42). *In vivo*, in a leukemia cell line (NB4) xenograft model treated with the anti-cancer agent selenite, tumors show a reduction of LC3-II and an increase of p62/SQSTM1, which is indicative of autophagy inhibition. Concomitantly, there is activation of caspase 3, indicative of apoptosis induction. In this model p53 signals to induce eIF4E dephosphorylation, preventing the binding of ATF4 to *MAP1LC3B* promoter and hence hampering autophagy (42). In contrast, in a study of selenite treatment of T-cell acute lymphoblastic leukemia, which are p53-deficient, eIF4E is phosphorylated and ATF4 mediates *MAP1LC3B* transcription, leading to an increase of autophagosomes. In this case apoptosis follows autophagy activation (41).

Initially, it was thought that transcriptional regulation of *ATG* genes depends entirely on the PERK/eIF2alpha/ATF4 axis, since upon ER stress, starvation or viral infection of cells bearing an eIF2alpha mutation non-responsive to PERK are incompetent to induce autophagy (62, 65, 66). However, a recent work with a functional assay to evaluate autophagosomes formation as well as cargo degradation, showed that ATF4 indeed induces the transcription of *ATG* genes involved in the formation of autophagosomes, but independent of PERK. PERK activates autophagy at steps subsequent to cargo sequestration in a transcriptional-independent way (61). Although these distinct roles could be cell- or context- dependent, it is important to consider them.

Although it keeps the name, eIF5A acts at the translation elongation phase. It alleviates translational stalling of the ribosome at hard-to-translate motifs. eIF5A enhances *ATG3* mRNA translation, which enhances autophagosome formation, as ATG3 is an E2-like protein necessary for LC3 (and other family members like GABARAP) lipidation. eIF5A has a unique aminoacid, hypusine, formed by post-translational modification of a conserved Lysine residue that is important for ribosome binding and translation. Hypusination of eIF5A is also necessary for autophagy induction (43).

Depletion of members of the scaffold eIF4G protein family, such as eIF4G1 and eIF4G2 (44, 45), or the RNA helicase eIF4A3 (43) cause an accumulation of autophagosomes, but it is still necessary to determine whether this is due to stimulation of autophagy or an impairment of the autophagic flux.

### Ribosomal Proteins Control Autophagy

**Figure 5:**
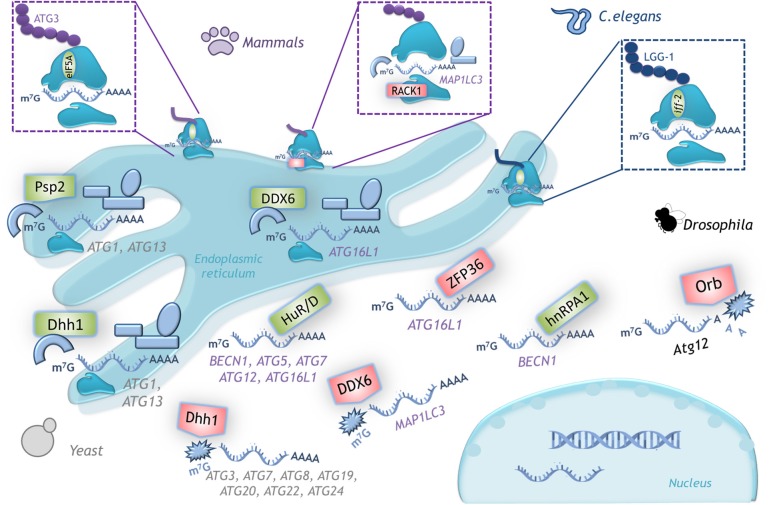
Examples of translational control of *ATG* mRNAs with conserved function in several organisms. A schematic representation of the translation factors that regulate positively (green) or negatively (red) translation of the indicated mRNAs. In yeast, Dhh1 either promotes or represses *ATG* mRNA translation according to the cell nutritional status. In mammals the dual function of DDX6 (Dhh1 homolog) is conserved. RNA binding proteins HuD, HuR and hnRPA1 are positive regulators and ZFP36 is a negative regulator of translation of the indicated mRNAs. The ribosomal protein RACK1 limits LC3 translation, while eIF5A-hypusine targets *ATG3* mRNA to favor autophagosome formation. In *C. elegans iff-2* (eIF5A homolog) is also a positive autophagy regulator. In *Drosophila* Orb promotes deadenylation and decay of its target mRNA. (See text for details).

RACK1 is a ribosomal protein component of the 40S subunit that promotes the formation of the 80S ribosome to allow translation. Depletion of RACK1 triggers autophagy induction in tumor-derived cell lines from breast, liver, connective tissue, and bone. Thus, RACK1 is a negative regulator of autophagy; this function depends on its localization at the ribosome, since a mutant unable to bind to the ribosome promotes *MAP1LC3B* mRNA-specific translation (46) (Figure 5).

Ribosomal protein RPS27L is also a negative regulator of autophagy. However, the mechanism to prevent autophagy is rather related with an upstream signaling that regulates the activation of autophagy. mTORC1, the main inhibitor of autophagy, is negatively regulated by DEPTOR. In the absence of RPS27L, DEPTOR is stabilized leading to its accumulation, inhibiting mTORC1 activity. Interestingly, RPS27L is reduced in human breast cancer cells compared with adjacent healthy tissue, perhaps having its reduction a promoting role during breast tumorigenesis (47).

### RNA Binding Proteins Control Autophagy

The Hu family of RNA binding proteins is effector of several post-transcriptional process of RNA metabolism, ranging from splicing to translation (67, 68). Hu family is composed of four members: HuR, HuB, HuC, and HuD. Interestingly, at least HuR regulates many processes such as inflammation, differentiation, migration, cell death, and as recently found, autophagy (50, 51).

Several *ATG* mRNAs coding for key proteins involved in initiation or elongation phases of autophagy are targets of Hu (Figure 5). In non-cancerous and cancerous human liver cells HuR depletion impairs the autophagic flux, with cells having smaller autophagosomes and lysosomes. By ribonucleotide immunoprecipitation it was demonstrated the interaction of HuR with *ATG5, ATG12*, and *ATG16* mRNAs; HuR binds to AU-rich elements (AREs) located at the 3'UTR of these mRNAs (50). That HuR enhances *ATG16* mRNA translation was also demonstrated in intestinal epithelium cells *in vitro* and *in vivo* in a mice line with intestinal epithelium-specific ablation of HuR (*IE-HuR*^−/−^); human intestinal mucosa from patients with Inflammatory Bowel Disease exhibit decreased levels of both HuR and ATG16L1, this is an interesting finding since autophagy is frequently defective in those patients (53). HuR induction of *ATG7* and *ATG16* mRNA translation was demonstrated in renal proximal tubular cells during hypoxia-induced autophagy; HuR binds to motifs located within *ATG7* mRNA coding region (51). *BECN1* mRNA also poses AREs at its 3′UTR, and upon starvation HuR stimulates BECN1 translation in non-cancerous keratinocyte, in breast and prostate cancer cells (52), and in human and rat liver stellate cells (49). *BECN1* mRNA translation is also enhanced by RNA binding protein hnRPA1 in human colon cancer cells (55). HuD also induces translation of *ATG5* mRNA. In pancreatic β cells silencing of HuD decreases *ATG5* mRNA translation, and conversely, HuD overexpression enhances *ATG5* mRNA translation (48).

Translational regulation of *ATG* mRNAs by RNA binding proteins is not always positive. The RNA binding protein ZFP36/TTP acts as a negative regulator of *Atg16* mRNA translation during ferroptosis, a type of cell death mediated by autophagy. ZFP36/TTP binds to AREs located at 3′UTR of *ATG16* mRNA and recruits deadenylation and degradations factors (54).

The examples of autophagy regulation by modulating *ATG* mRNAs translation reviewed above refer to conditions found in tumor environment, such as hypoxia and starvation. In some situations autophagy induction favors cancerous cells survival, for example in response to starvation (52) or hypoxia (51), while in other situations autophagy is rather inhibited to evade cell death (49). It is currently unknown what regulates the binding of Hu proteins to target mRNAs. Recently, it was reported that the circular RNA *circPABPN1* blocks the interaction between HuR and *Atg16 mRNA* (53). Whether other Hu/mRNA interactions are also regulated by circRNAs or other mechanisms, such as post-translational modifications (69), or whether it is constitutive under certain circumstances, need to be further studied.

### Translational Control of Autophagy in Other Organisms

Autophagy is an evolutionarily conserved process, therefore, it is reasonable to think that its regulation is also conserved across species. Here we review some examples (Figure 5).

During *Drosophila* oogenesis, protein Orb negatively modulates translation of *Atg12* mRNA, and thus autophagy (56). Orb belongs to a highly conserved RNA-binding protein family that recognizes cytoplasmic polyadenylation elements located in the 3′-UTR, and can both upregulate or downregulate its target depending on its association with polyadenylases or deadenylases, respectively. Several other autophagy mRNAs also contain cytoplasmic polyadenylation elements (*Atg1, Atg2, Atg5, Atg7, Atg8a*, and *Atg18*), suggesting that Orb might control autophagy at different steps. It has not yet been investigated whether members of the CPEB-family, orthologs of Orb in vertebrates, maintain this regulation. It would be interesting to study if under stress conditions CPEBs associate with polyadenylases to induce autophagy instead of repressing it.

In yeast there is an autophagy regulator with a dual role that can either repress or promote the translation of *ATG* mRNAs, depending on the nutritional status. The RNA helicase Dhh1 under nutrient replete conditions acts as a negative regulator of *ATG* mRNAs that code for proteins participating in almost all stages of the autophagic pathway: initiation (Atg20, Atg24), elongation (Atg3, Atg7, Atg8, Atg19), and recycling (Atg22) (57). Unexpectedly, under nitrogen starvation-conditions Dhh1 switches its function to become a positive regulator of autophagy, and promotes the translation of *ATG1* and *ATG3* mRNAs (58). In mammalian cells there is an ortholog of Dhh1 known as DDX6 that conserved this dual role, however the mRNAs targets are different (57, 58). Also in yeast, the RNA-binding protein Psp2 is a positive translational regulator of autophagy. Under nitrogen-starvation, Psp2 binds the eIF4E/eIF4G complex and the 5′-UTR of *ATG1* and *ATG13* mRNAs to promote their translation (70).

The positive relationship between eIF5A and autophagy stimulation is also conserved in *C. elegans*. Worms deficient on *iff-2* (eIF5A homolog) show a decreased punctate pattern of the GFP::LGG-1 (an LC3 ortholog) fusion protein (43). Considering that protein translation integrates signaling from a wide variety of stimuli, to couple autophagy regulation with protein synthesis is essential.

## Targeting Translation and Autophagy in Cancer

Traditionally, most studies on cancer have focused on the processes occurring at the DNA level, such as mutations and chromosomal rearrangements, variation in chromatin methylation, and transcriptional dysregulation. In the last years, new evidence has emerged supporting the notion that cancer may also result from failures in the interconnection among different metabolic networks and molecular processes that underlie even disparate diseases (1). Studies on the interplay between translation and autophagy have led to the identification of new molecules that can be chemically targeted with clinical implications in the treatment of several types of cancer. Here we mention few examples.

### Targeting the PERK/eIF2alpha/ATF4 Axis

Recently, the PERK/eIF2alpha/ATF4 axis has been involved in the onset and development of different types of cancer. For example, ER stress-mediated PKR activation regulates the induction of autophagy during tumorigenesis in carcinoma, gastric adenocarcinoma, and melanoma cells. When PERK is inhibited either pharmacologically with the drug GSK2606414 or genetically by using siRNA to silence PERK expression, decreased both LC3 expression and LC3-II lipidation (71). Additional examples of autophagy induction by the PERK/eIF2alpha/ATF4 axis in different cancer models are summarized in Table 3.

**Table 3:** Autophagy induction by the PERK/eIF2alpha/ATF4 axis in different cancer models.

**Neoplasia**	**Cells/model**	**Inducer**	**Reference**
Glioblastoma	Multiple human glioblastoma cells	Melanoma differentiation associated gene-7/interleukin 24 (GST-MDA-7/IL-24)	(72)
	U87MG	Glucosamine-induced ER stress	(73)
	Primary glioblastoma human multiforme cells	Recombinant Melanoma differentiation associated gene-7 (*mda-7*) adenovirus (Ada. *mda-7*)	(74)
Ovarian cancer	Epitelial human Pa-1 cells	Metformin-induced ER stress	(75)
Breast cancer	Human MCF-7 cells	Ursolic acid-induced ER stress	(76)
Neural radiation myelitis (spinal metastasis)	Banna mini-pigs spinal cord cells	Iodine-125-induced ER stress	(77)
Leukemia	Human acute promyelitic leukemia NB4 cells	Selenite-induced ER stress	(42)
Bone cancer	Human osteosarcoma MG63 and KHOS cells	2-methoxyestradiol	(78)
	Human osteosarcoma MG63 cells	Thapsigargin-induced ER stresses	(79)
MYC-induced tumorigenesis	Human B-cell lymphoma P493-6B cells and mouse embryonic fibroblast	c-Myc-induced ER stresses	(80)

### The Akt/mTOR Pathway

Research in different kinds of cancer has focused mainly on mTOR or the Akt/mTOR pathway (81–84), which are signaling cascades shared between translation and autophagy. Here we review examples of molecules currently tested targeting this pathway (squematized in Figure 4).

Studies in glioma cells have shown that celastrol possess antitumor effects. It inactivates mTOR, drives cell cycle G2/M phase arrest, autophagosomes accumulation apparently due to lysosomes impaired function, and apoptosis (85). Studies with rapamycin in various cancer cell lines showed that it increases the number of LC3 puncta suggesting autophagy induction (86, 87), but not apoptosis, and this effect is synergized in combination with PI3K or AKT inhibitors (86). However, neuroblastoma or squamous cell carcinoma seem to be resistant to autophagy induction mediated by rapamycin, apparently because RAPTOR maintains bound to mTOR, and these cells are sensitized only when they are treated with mTOR catalytic inhibitors (87). This finding suggests that using combined therapies could be more effective or even necessary to treat certain types of cancer.

The use of quercetin, a flavonoid present in fruits and vegetables, inactivates the AKT/mTOR pathway and induces HIF-1alpha signaling in gastric cancer, promoting simultaneously apoptosis and protective autophagy. In this case inhibition of autophagy reduces cell viability (88). Also in a study of breast cancer, quercetin reduced cell invasion, and migration by inactivating also Akt/mTOR pathway and leading to an apparently autophagy induction. It is interesting to note that the mechanism to reduce breast cancer cells migration could be due to a quercetin-reduced expression of matrix metalloproteinase 9, and this reduction is abrogated when autophagy is inhibited, suggesting a role of autophagy regulating metalloproteinases availability (89). Since autophagy machinery can also contribute to alternative secretion (90), autophagy could regulate metalloproteinasses maturation and/or secretion. This particular non-catabolic function of autophagy needs to be further investigated in cancer research. Since autophagy in these cases is induced in response to quercetin and favors tumor progression, a pharmacological combination with autophagy inhibitors could increase quercetine effectivity.

On the other hand, the Akt inhibitor 1L-6-hydroxymethyl-chiro-inositol 2(R)-2-O-methyl-3-O-octadecylcarbonate shows radiosensitizing effects in malignant glioma cells by apparently inducing autophagy, with an overall outcome of anti-tumorigenesis (91). Curcumin also inhibits the Akt/mTOR/p70S6K pathway and activates the ERK1/2 pathway, resulting in autophagy induction both *in vitro* and *in vivo*. In a subcutaneous xenograft model of U87-MG cells, curcumin induces autophagy and inhibits tumor growth (92).

A summary of compounds targeting translation and autophagy in cancer is presented in Table 4.

**Table 4:** Therapeutic compounds used for autophagy induction or inhibition in cancer.

**Compound**	**Target**	**Model system**	**Autophagy evaluation**	**References**
GSK2606414	PERK	Basal cell carciona (BCC/KMC1) Gastric Adenocarcinoma (AGS) Melanoma (A375) Imiquimod	↓LC3-II ↓EGFP-LC3-II puncta	(71)
Celastrol	mTOR	Glioma (U251, U87 and C6) Pre-treatment CQ	↑LC3, BECN1,p62 ↑LC3 puncta ≠LC3-II	(85)
LY294002 UCN-01 (7-hydroxystaurosporine)	PI3K Akt	Glioma (U87-MG, U373-MG and T98G) Rapamycin	**↑**MDC stain	(86)
Rapamycin RAD001 (rapalogue) KU-0063794 (catalytic mTOR inhibitor) WYE-354 (catalytic mTOR inhibitor)	mTOR	Bladder carcinona (RT112) osteosarcoma (U2OS) neuroblastoma (SK-N-SH) squamous cell carcinoma (HN10)	↑LC3 punctate (all treatments) ↑LC3 punctate (only with catalytic mTOR inhibitors)	(87)
AZD8055	mTOR	lung cancer H838 and A549 +E64d/pepstatinA	↑LC3-II ↑LC3 puncta ↑Acridine orange stain ↑↑LC3-II	(93)
Metformin	AMPK	Melanoma (A375, and SKMel28) Xenograft model	↑LC3-II, BECN1 ↑LC3 puncta ↑Autophagosomes (TEM) ↑ CatB activity ↑LC3-II ↑LC3 puncta	(94)
Metformin	mTOR	Esophageal squamous cancer cells (ESCC) Pre-treatment 3-MA or CQ Xenograft	↑LC3-II, BECN1 ↑GFP-LC3 puncta ↑ Autophagosomes (TEM) ↑ Acridine orange and MDC stain ↓LC3-II, BECN1 ↓LC3-II, ↓p62 (IHC)	(95)
Akt inhibitor (1L-6-hydroxymethyl-chiro-inositol 2(R)-2-O-methyl-3-O-octadecylcarbonate)	AKT	Glioma U87-MG U87-MG Δ*EGFR*	↑GFP-LC3 punctate ↑autophagosomes (TEM), ↑Acridine orange stain	(91)
Curcumin	Akt/mTOR/p70S6K/4E-BP	Glioma U87-MG and U373-MG Xenograft	↑GFP-LC3 puncta ↑LC3-II ↑autophagosomes (TEM) ↑ Acridine orange stain ↑LC3-II, ↑LC3 (IHC)	(92)
Quercetin	Akt-mTOR	Breast cancer MCF-7 and MB-231	↑LC3-II, LC3 puncta	(89)
Quercetin	Akt-mTOR	Gastric adenocarcinoma AGS and MKN28	↑ LC3-II, BECN1, ATG7, ATG5/12 ↑ GFP-LC3 puncta ↑ Autophagosomes (TEM) ↑ Acridine orange stain	(88)
AZD1208	pan-PIM kinase inhibitor	Chronic lymphocytic leukemia (CLL) BafA1	↑Acridine orange stain, ↑LC3-II ↑↑LC3-II	(96)
Parthenolide	Oxidative stress downregulation of 4E-BP1	See Table 2	See Table 2	(38)
Selenite	eIF2alpha phosphorylation by ROS or ER stress	See Table 2	See Table 2	(41, 42)
Erastin	Ferroptosis inducer	See Table 2	See Table 2	(49, 54)

## Concluding Remarks

A common feature of cancerous cells is having aberrant translation, as many oncogenes and tumor suppressors affect the translation machinery. Many translation initiation factors are dysregulated in various cancers, and increased levels of eIF4F complex render cancer cells resistant to chemotherapeutics (7). Considering also that protein synthesis is coupled to autophagy regulation, targeting translation factors is a promising therapy that could at same time reduce autophagy induction. Nevertheless, as reviewed above, even though in early tumor environments under hypoxia and low nutrients availability autophagy induction favors cancerous cell survival, in other cancerous cells autophagy is rather inhibited to evade cell death. Therefore, it is not possible to generalize the use of autophagy inhibitors to treat cancer. A characterization of the function of autophagy in particular types of cancer is necessary.

Once the specific function of autophagy is known, targeting autophagy machinery to modulate its function could complement chemotherapy to increase its effectiveness. Most recently, novel strategies to treat cancer have been developed that utilize nanoparticles to target mTOR and AMP-activated protein kinase (AMPK) pathways. These nanoparticles, made up of different metal or silica materials, are designed to overcome obstacles usually encountered with traditional drugs, such as low specificity, irregular distribution in tissues and organs, rapid drug clearance, and biodegradation. The clinical relevance of these innovative therapies is currently being evaluated (97).

## Author Contributions

GH conceived the manuscript, gathered information, and wrote part of the paper. PA-R gathered information, wrote part of the paper, assembled Tables 1, 2, 4, and did Figures 2–5. GM gathered information, and contributed to assemble Tables 2–4, and did Figure 1. SC-O gathered information, wrote part of the paper, and integrated the information.

### Conflict of Interest

The authors declare that the research was conducted in the absence of any commercial or financial relationships that could be construed as a potential conflict of interest.
